# Amyotrophic Lateral Sclerosis as a Systemic Disease

**DOI:** 10.3390/ijms24087083

**Published:** 2023-04-11

**Authors:** Viviana Moresi

**Affiliations:** Institute of Nanotechnology, National Research Council (CNR-NANOTEC), Sapienza University of Rome, 00185 Rome, Italy; viviana.moresi@cnr.it

The goal of this Special Issue is to report new research progress and reviews concerning amyotrophic lateral sclerosis (ALS). Patients with ALS diagnosis are clinically highly heterogeneous, based on either genetic or sporadic (unknown) origins, and they manifest different forms (spinal motor neuron injury or bulbar paresis) and survival rates, ranging from months to decades. Unveiling pathogenetic mechanisms in different tissues/cell types and finding reliable markers are high priorities for the development of treatments for ALS.

Potential therapeutic targets came from [1,2,3]. Specifically, an important role for Connexin 30 (Cx30) in promoting astrocyte inflammation in ALS has been addressed in [3], pointing to the targeting of the gap junctions/hemichannels as novel therapeutic targets for treating ALS. Moreover, alterations in the expression of circular RNAs and circular RNA–microRNA–mRNA interactions in spinal cords of ALS patients have been found in [2], providing evidence for potentially unexplored targets for the treatment of ALS. Another potential target has been identified in granzyme A, since a partial deficiency of this regulator of the immune response is protective against the progression of the disease by decreasing the expression levels of inflammatory and oxidative stress markers in spinal cords and skeletal muscles in a murine model of ALS [1]. A significant contribution to understanding how ALS affects skeletal muscle metabolism comes from [4]. In this study, the authors defined the dysfunction of lactate metabolism in the skeletal muscles of ALS mice by quantifying the amount of NAD+ and NADH nucleotides and the activities of the NADH transport enzymes (the malate–aspartate shuttle system). Swim training partially reverses changes in LA metabolism in ALS murine skeletal muscle by influencing the glycolytic enzyme and malate dehydrogenase activities, thus improving skeletal muscle mass by adjusting the energy demands. In addition, a step toward precision medicine in ALS has been performed by the study of Renzini A. et al. [5]. In this paper, the authors examined sex differences in ALS onset, progression, and lifespan in SOD1^G93A^ mice, finding a global sex-dependent effect on disease onset and mouse lifespan. Moreover, the protective role of histone deacetylase 4 (HDAC4) in skeletal muscles in female SOD1^G93A^ mice was confirmed, even though it has a more dramatic effect in males. Together with the motoneurons and skeletal muscles, other tissues and cell types are involved in ALS: interestingly, the sensory and autonomic systems are also involved, as reviewed in [6].

The impact of the transmembrane protein 106B (TMEM106B) gene on disease susceptibility and genotype–phenotype correlation in a cohort composed of 865 ALS patients has been investigated [7]. Interestingly, the effects of the single nucleotide polymorphism rs1990622 in TMEM106B included both cognitive, such as language, and verbal fluency, and they also include memory and motor functions and, consequently, the functional status of ALS patients. Considering that the presence of cognitive and behavioral impairment is a prognostic factor in ALS and that genetic variability influences the patient’s survival when administering novel treatments, TMEM106B should be considered as a susceptibility factor in ALS. Similarly, in [8], the authors explored the functional consequences of a missense mutation (R573G) located in the dimerization domain of the TANK binding kinase 1 (TBK1), a kinase involved in different pathways, including the immune response and autophagy. R573G TBK1 mutation impaired the protein kinase activity, leading to increased phosphorylation and cleavage of the TDP-43 protein, together with enhanced ROS levels and increased expression of IL-6, recapitulating some of the pathological features of ALS. The accumulation of TDP-43 aggregates in the central nervous system, which is a common feature of many neurodegenerative diseases, including ALS, and a study of the molecular properties of TDP-43 protein aggregation in droplets has been performed [9] to provide information to design ligands to modulate the protein phase behaviors. Most of the mutations of TDP-43 associated with ALS are in the low-complexity domain. By screening different conditions, the authors found the conditions to finely tune the interactions affecting the protein liquid–liquid phase separation equilibrium and droplet sizes of the TDP-43 low complexity domain.

Searching for new immune mediators in ALS progression and prognosis, Picher-Martel V. et al. identified a distinct plasma immune profile in sporadic ALS patients, similar to two murine models of ALS, i.e., SOD1^G93A^ and UBQLN2^P497H^; this was also observed in TDP-43^G348C^ transgenic mice, and this is involved in adipocyte function and leptin signaling [10]. Additionally, a SOMAscan proteomic analysis identified 42 proteins whose levels significantly differ between the plasma of 16 ALS patients and eight healthy controls [11]. Four of these proteins—i.e., TARC/CCL17, TIMP-3, NID1 and NID2—that were significantly upregulated in the patient’s sera, support the existence of a ECM–chemokine interplay in the ALS pathogenesis that may be potentially targeted by therapies.

Finally, with the aim to improve the current diagnostic criteria for an earlier and more efficient diagnosis of ALS, a review summarized and discussed the potential of machine learning (ML)-based magnetic resonance neuroimaging methods for a patient’s diagnosis and follow up [12].

Overall, the studies presented in this Special Issue will contribute to the scientific community to the common goal of finding a more effective treatment for an earlier diagnosis and counteracting the progression of this still lethal neurodegenerative disease.
